# A qualitative study of university students’ perspectives of hope during the COVID-19 pandemic

**DOI:** 10.1177/20503121231185014

**Published:** 2023-07-23

**Authors:** Aria Keshoofy, Konrad Lisnyj, David L Pearl, Abhinand Thaivalappil, Andrew Papadopoulos

**Affiliations:** 1 Department of Population Medicine, University of Guelph, Guelph, ON, Canada; 2 Family Health Division, City of Hamilton Public Health Services, Hamilton, ON, Canada

**Keywords:** Campus, COVID-19, epidemiology/public health, hope, mental health/psychiatry, qualitative

## Abstract

**Background:**

Students in higher education commonly experience mental health problems. There is an ongoing need to explore potential intervention targets to focus on mental health promotion among students. Hopefulness may alleviate or be protective against various negative mental health conditions such as depression, anxiety, suicide, and trauma-related disorders.

**Objective:**

To explore postsecondary students’ meanings and experiences of hope during the COVID-19 pandemic and identify factors affecting hopefulness during crises.

**Methods:**

Purposive sampling was used to recruit participants for online semi-structured interviews in a university located in Southwestern Ontario, Canada. Data were analyzed using thematic analysis.

**Results:**

In total, 12 participants were interviewed, and 4 themes were generated: (1) hope is a complex concept with an associated set of behaviors, (2) cognitive framing of hope as a means of student resilience, (3) COVID-19 as an antagonist which amplifies preexisting student concerns and issues, and (4) the social and physical environments serve as barriers and enablers to hope and well-being. Hope was perceived as a positive mental trait, external events and the environment were reported to impact hope, and those who were generally more hopeful adjusted better mentally when unexpected circumstances arose.

**Conclusions:**

Findings shed light on the interconnectedness and complex nature of hope, its sources, and enablers. Novel findings include the ways in which hope was affected during the COVID-19 pandemic. Recommendations for individual- and community-based interventions include targeting enablers to hopefulness by promoting social support systems, offering virtual extracurricular activities, and delivering alternative approaches to teaching and learning.

## Introduction

Postsecondary student mental health has been an emerging topic of interest in recent years. Students in higher education contexts commonly experience mental health problems because of factors related to emerging adulthood—they move away from home, develop new relationships, take on increased academic workloads, gain employment, and have additional responsibilities.^1,2^ Data between 2013 and 2022 show increase in prevalence of mental health challenges such as depressive symptoms, anxiety, and loneliness.^3–6^ Moreover, the Center for Collegiate Mental Health reported an increase in symptoms of student distress, generalized and social anxiety, and depression over the last 11 years.^7^ To add to this already highly impacted group, the COVID-19 pandemic amplified ongoing mental health challenges among postsecondary students.^7–9^ In 2021, nearly 72% of students sought mental health services citing the pandemic had negatively affected their mental health.^7^ A cohort study from 2020 to 2021 found increase in symptoms of depression, anxiety, insomnia, and self-harm among Canadian undergraduate students.^10^ These findings highlight the need for more robust early identification and intervention efforts among university and college students.

Canadian postsecondary students who self-reported experiencing hopelessness increased from 54% to 79% between 2013 and 2022.^3–6^ Some researchers have advocated for hope-based interventions among postsecondary students because of its potential to improve domains in mental health (e.g., resilience, depression) and academic performance.^11,12^ Hope theory posits that all individual actions are motivated by the pursuit of goals.^13^ The theory focuses on positive goal-related outcomes with an emphasis on pathway cognition (i.e., the ability to identify routes to one’s goals) and agency (i.e., the motivation to pursue these pathways).^13^ It is theorized that throughout life, individuals have a great number of goals that vary in terms of their temporal frame, specificity, and difficulty. Hopeful individuals, commonly referred to as “high-hope individuals,” differ from their less-hopeful counterparts in many ways.^13,14^ Most notably, they differ in their pathway cognition and agency.^13^ Even during circumstances when individuals are impeded on their way to their goal, high-hope individuals produce plausible alternative routes to the same goal compared to their low-hope counterparts.^13^

Due to the increased mental health problems observed during the COVID-19 pandemic,^10,11,15^ there is a timely need to revisit strategies focusing on early intervention and coping strategies to better serve postsecondary students, prepare them for the challenges associated with emerging adulthood, and coping during crises.^1^ Previous research among postsecondary students have found that feelings of hopelessness significantly increase the likelihood of reporting anxiety affecting academic performance,^16^ and in contrast, hope is a strong predictor of life satisfaction.^17^ It is believed that hopeful individuals have advantages academically, socially, and in other aspects of their lives. Consequently, there may be several benefits if postsecondary institutions introduce campus-wide interventions aimed at bolstering and developing students’ sense of hope as there is some evidence of hope-integrated interventions improving clinically significant mental health symptoms such as depression, anxiety, and trauma.^18,19^ Seeing as hope may be a promising area to address among this priority population, a survey of over 11,000 Canadian postsecondary students found 79% reported feeling some level of hopelessness during the COVID-19 pandemic in 2022.^4^ While there is an emphasis placed on the treatment of mental health issues on campuses, research gaps exist surrounding what precipitates hopelessness in these settings. Some research studies investigating peer mentorship programs and goal-directed thinking have demonstrated improvements to student mental health, sense of community, emotional support, motivation, and satisfaction,^20–22^ suggesting these avenues may be feasible as intervention targets for improving well-being outcomes among students. Despite knowing that students who feel isolated are at greater risk of hopelessness,^23^ factors associated with hopelessness or how they manifest in this condition have not been comprehensively studied. Thus, the aims of this study are to explore how university students perceive and make sense of hope during the COVID-19 pandemic and identify the factors affecting hopefulness among this priority group.

## Methods

### Study design

This study used a qualitative descriptive design to provide insight to the phenomenon of hope through subjective experiences.^24^ We conducted one-on-one semi-structured online interviews with university students. This method was applied to facilitate open-ended responses, promote two-way communication, and to uncover in-depth perspectives on sensitive topics surrounding student mental health. Participants were recruited and interviewed by the first author to investigate students’ subjective meanings of hope, how hope was affected during COVID-19 pandemic, and its connection to student mental health on campus. This study was carried out at an English-speaking, public postsecondary institution in Southwestern Ontario, Canada.

### Study setting

This study took place at a large English-speaking university located in Ontario, Canada. The institution has approximately 29,000 full-time equivalent students. The study took place between May and August of 2021 during the COVID-19 pandemic. Due to restrictions and preventive measures associated with the COVID-19 pandemic, the study and data collection was conducted entirely virtually.

### Participant recruitment

Purposive sampling was used to recruit participants and interviews were conducted between May and August 2021. Participants were recruited through varsity sports teams, undergraduate volunteers, and the student wellness center which distributed the research team’s letter of information via email. This study was part of a larger project exploring a peer mentorship program at the participating university, and we used these channels for participant recruitment particularly because of greater engagement, response rates, and lived experience in these groups could offer regarding the topic of investigation. Due to nature of the recruitment process where individuals were asked to share the recruitment email with their formal and informal networks, we were unable to ascertain how many individuals the recruitment was sent to and by extension, also the response rate.

Interested participants contacted the first author via email and were given a letter of information and informed consent form to complete. Participants were provided a $10 electronic gift card of their choosing in appreciation of their time. There is no consensus on the minimum required sample size for qualitative studies.^25,26^ Instead, some researchers propose critically evaluating aspects of data adequacy and data saturation as well as considering the context and topic of investigation.^25,26^ Therefore, our study followed a similar approach for data collection until no new information or themes were observed in the data. To go beyond this and confidence in the adequacy of data, we employed a stopping criterion of 3, which indicates the minimum number of additional interviews to be conducted after achieving data saturation.^27^ Recruitment and interviews were carried out until data saturation was achieved, resulting in 12 participants being interviewed.

### Data collection

Through a series of consultations with the research team and service providers, an interview guide was developed. The guide contained 15 questions with a set of probing questions related to hopefulness, mental health, COVID-19, and campus activities (e.g., “What comes to mind when you think of the term hope?,” “Would you describe yourself as a hopeful person?”). These questions were pre-tested among members of the research team and two university students, and the interview guide was subsequently amended. All interviews were conducted by the first author, in a one-on-one online setting using Microsoft Teams (Microsoft, Redmond, Washington). Interviews ranged from 20 to 60 min, were audio recorded, and later transcribed. To enhance trustworthiness of findings, participant transcripts were handed back to the respective participants to allow individuals an opportunity to verify, correct, and expand on any opinions expressed during the interview.^28^

An online demographic questionnaire was shared with participants after the interviews were completed. The questionnaire contained 12 items related to demographic characteristics (e.g., “What is your gender identity?”) and frequency of mental health outcomes (e.g., “Have you ever been diagnosed with depression?”). These questions were taken verbatim from a previously validated and reliable survey.^3,29^ The full interview guide and questionnaire are provided as Supplemental Material.

### Data analysis

For quantitative data, descriptive statistics in the form of frequencies and proportions of all demographic data were generated using Microsoft Excel (Microsoft, Redmond, Washington, USA). For qualitative data, thematic analysis was conducted by the first author using a semantic approach and a mixture of inductive and deductive coding. We anticipated some recurring features by consulting with researchers on the team and other experts with content expertise in mental health. Codes were developed to reflect this, and new codes were inductively generated for unanticipated features presented during the data familiarization and coding process. Subsequently, codes were reviewed and revised by the remaining members of the research team. During this process, codes were combined, deleted, and amended before a final set of codes were generated. Throughout the analysis, we leaned on reflexive thematic methods which allows for an open coding process, inductive or deductive coding, and an iterative generation of themes.^30^ Each transcript was reviewed repeatedly during the analysis, and illustrative quotes were extracted to support each finding. Participants were assigned pseudonyms to protect anonymity and humanize their perspectives. Preliminary themes were refined through discussion and peer debriefing with the research team until the final set of themes were generated. Analysis was conducted using NVivo 1.6.1 qualitative analysis software (QSR International, Doncaster, Australia). All steps were conducted by the first author and reviewed and refined by the research team through discussion.

### Ethics

Informed consent was collected in writing once during the recruitment phase, immediately after the participant read through the letter of information, and once again verbally prior to starting the interview. To protect anonymity, all participants were assigned pseudonyms. The institutional research ethics board at the University of Guelph granted ethics approval to carry out this study (REB #20-12-015).

## Results

In total, 12 participants were interviewed, but 2 participants did not complete the demographic survey component. Those who responded to the survey were mostly women (70%), described their health as very good (60%), had a B or greater cumulative grade point average (GPA) (80%), reported feeling very lonely in the past year (80%), and did not report traumatic financial stress (100%), traumatic academic stress (30%), never had suicide ideation (70%), or ever attempted suicide (90%) (Table 1).

**Table 1.** Participant responses to the demographic component of the hopeful thinking study (*n* = 10).

Question	*N* (%)
Gender	
Man	3 (30)
Woman	7 (70)
How would you describe your general health?	
Very good	6 (60)
Good	3 (30)
Fair	1 (10)
Excellent, poor, or don’t know	0 (0)
What is your approximate cumulative GPA?	
A	5 (50)
B	3 (30)
C, D, or F	0 (0)
N/A	1 (10)
Choose not to respond	1 (10)
What is your college?	
Arts	4 (40)
Biological science	3 (30)
Engineering and physical sciences	1 (10)
Social and applied human sciences	1 (10)
Ontario agricultural college	1 (10)
In the last 12 months have you ever felt very lonely?	
No, not in the last 12 months	2 (20)
Yes, in the last 2 weeks	1 (10)
Yes, in the last 12 months	7 (70)
Never, “Yes, in the last 30 days,” or choose not to respond	0 (0)
In the last 12 months have you ever seriously considered suicide?	
No, never	7 (70)
No, not in the last 12 months	1 (10)
“Yes, in the last 2 week” or “Yes, in the last 30 days”	0 (0)
Yes, in the last 12 months	1 (10)
Choose not to respond	1 (10)
In the last 12 months have you ever attempted suicide?	
No, never	9 (90)
No, not in the last 12 months	1 (10)
“Yes, in the last 2 weeks,” “Yes, in the last 30 days,” “Yes, in the last 12 months,” or “Choose not to respond”	0 (0)
Have you ever been diagnosed with depression?	
No	5 (50)
Yes	5 (50)
In the last 12 months has the following been traumatic or difficult for you to handle: *Financial stress*?	
No	10 (100)
Yes	0 (0)
In the last 12 months has the following been traumatic or difficult to deal with: *Academic stress*?	
No	7 (70)
Yes	3 (30)
In the past month, how often did you have the feeling that you belonged to a community like a social group or your neighborhood?	
Never	0 (0)
Once or twice	2 (20)
About once a week	1 (10)
About two or three times a week	3 (30)
Almost everyday	2 (20)
Everyday	2 (20)

Two out of the 12 participants did not complete or submit the demographic survey.

Thematic analysis resulted in the generation of four themes: (1) hope is a complex concept with an associated set of behaviors, (2) cognitive framing of hope as a means of student resilience, (3) COVID-19 as an antagonist which amplifies preexisting student concerns and issues, and (4) the social and physical environments serve as barriers and enablers to hope and well-being. Themes and supporting illustrative quotes are presented below.

### Theme 1: Hope is a complex concept with an associated set of behaviors

Students linked hope to ideas closely tied to optimism, positivity, expectations of positive outcomes, ambition, success, and wishing for the best in the future:

. . . being able to look forward to something. Being hopeful is kind of like a headspace where you can appreciate that things will either get better or that good things will happen eventually. (Participant 10, Mark)

Most participants identified a direct relative who most closely resembled hope. The character traits included perseverance, determination, and a positive outlook. Hopefulness was perceived as an intrinsic, lifelong value, even during times of struggle:

. . . my aunt she’s gone through a lot, but she went through really bad divorce. Life really hasn’t gone according to plan so she’s someone that shows a lot of hope. Also, my grandpa has lung cancer, and he just keeps on kicking; he has a lot of hope. (Participant 9, Jessica)

Participants felt a hopeless person was one with little motivation, depressed, had a low mood, lacked in purpose, gave up easily, was easily overwhelmed, expected failure, was isolated, tended to overthink, seemed removed, and was quite limited socially:

I think what I think of is a person who lays in bed all day, has their curtains drawn, doesn’t really seem to be motivated by much, constantly saying stuff like, “Oh I hate my life. There’s nothing to look forward to.” Someone very “mopey” and down all the time even if something good happens. (Participant 7, Blake)

[A] person who’s hopeless probably doesn’t have goals or aspirations. I think they would definitely be someone who feels very stuck and like they aren’t necessarily moving. (Participant 10, Mark)

### Theme 2: Cognitive framing of hope as a means of student resilience

Participants referred to future thinking, identifying alternative routes to the same goals, or even engaging in being flexible with their goals to better meet their new expectations. Hopeful participants emphasized areas within their volition and reminders on the transient state of negative events:

I think a lot of my hope just comes from acceptance that I don’t have control over the situation, and I should just focus on what I do have control over. Which at the end of the day is my attitude that I set every morning. I wake up and every time I’m faced with a decision to make, it’s just the attitude that I bring forward towards it [that I have control over]. (Participant 4, Stephen)

Whenever there is a stressful situation, I just think like, “It’ll be done in a week” or I always sort of think like “All you can do is your best and that’s really it.” Try your hardest if you don’t do a good job then that’s OK, it’s not the end of the world, it’s okay. (Participant 9, Jessica)

### Theme 3: COVID-19 as an antagonist which amplifies preexisting student concerns and issues

Most participants stated they were generally hopeful individuals in life. However, individuals reported feelings synonymous with hopelessness when asked about the impact that being a student had on their mental health:

I definitely wouldn’t say it has been like that good for my mental health. I think overall in undergrad we’re expected to handle just a pretty challenging workload. I think it is at times unreasonably difficult. And also, I would say my biggest stress is school related, but it is more so surrounding the fact that obviously like my GPA is permanent. (Participant 1, Emma)

. . . most professors are accommodating, but sometimes they’re not because they don’t get it and they didn’t play sports growing up. Which is tough, but I think the worst part is when you have a prof who doesn’t understand. We went to [a tournament] last year, and I had to write a midterm there, and I got a very poor mark that wasn’t fun because I was like “What do you want me to do?” (Participant 7, Blake)

In addition to the regular stressors associated with higher education, students described the amplified challenges experienced during the COVID-19 pandemic such as the transition from in-person to remote course delivery:

Definitely the social aspect changed the most . . . yeah definitely [impacted mental health]. I think when it was like the heavier lockdowns, where I was like can’t leave the house, can’t see friends, can’t see family, don’t have rugby, everything was online, it was just like a really tough time to like compared to what life was like before like where everything was just so on track, and then everything just like flipped. (Participant 6, Zoe)

The first lockdown, I moved back in with my parents, and we didn’t know how long it was going to last, and then in the summer kind of the point that I was just feeling pretty shitty was when I realized it was going to be a long, long time. Not just the next three months. We won’t be able to get back to normal life for like a year, and I don’t have friends around, and I don’t have anything going on so that’s when things were like I would say at the worst even though that wasn’t at the height of the lockdown. (Participant 8, Joshua)

### Theme 4: The social and physical environments serve as barriers and enablers to hope and well-being

Participants reported that belonging to a community, such as a team, a club, or a job improved hopefulness and well-being. In contrast, the adjustment caused by changing COVID-19 restrictions along with a reduction in hope led to languishing mental health:

Around last year October to November . . . my mental health had been at its worst, since ever. I haven’t even told my parents about it. I started cutting late last year, like—it’s embarrassing. I haven’t told anyone . . . I did get laid off. I worked at a restaurant as a waiter so that did impact how many times I would go out per week and my finances, and because of that my mental health a little bit. And the athletics center closed, so I couldn’t play badminton or go swimming with my friends. Yeah, I really miss badminton. (Participant 3, Nicole)

Most participants connected the physical environment to peers, roommates, and shared social activities. These opinions highlighted the salience of the campus context on normalcy and the university experience:

It was that structure [traditional style residence], but I was in an athletic cluster which made it a lot more community oriented. Like people spent a lot of time in the common area, people kept doors open a lot, people in the hallway a lot, it was like a good community there. (Participant 8, Joshua)

My roommate was fine. She was a nice girl. I like the whole experience of going to the library. I love that and I loved going to get food with other athletes in my friends, but I enjoyed [residence]. I didn’t get a major experience because I got sent home in like March so, yeah. (Participant 7, Blake)

## Discussion

This qualitative study aimed to explore students’ perceptions and meanings of hope, contextualize hope during the COVID-19 pandemic, and how hope plays a role in student well-being. We recognize that well-being is a nuanced topic that deserves several cognitive, affective, sociocultural, and environmental considerations. Our study, however, serves as an information-gathering step in moving toward student well-being with a focus on hope because of the negative nature of hopelessness on academic and personal outcomes of students.^16^

Participants in our study who identified themselves as being hopeful also spoke about coping strategies in response to negative events, emphasized what was within their control, and were flexible in goal setting. This is line with previous research that states high-hope individuals are flexible, think of several avenues to their goals, and remain persistent even under stressful conditions.^13,14^ Snyder (2002) found that hope tends to flourish relative to the probability of goal attainment.^14^ This was supported by our study as participants identified high levels of hope when they were accomplishing tasks which they believed would ultimately lead to larger goals being attained. In addition, Snyder (2002) theorized high-hope individuals appear to calibrate their goal expectations according to the relevant boundary conditions.^14^ When the study participants reflected on the disruptions they had experienced to achieving their goals, their immediate emotional response was disappointment. Yet, hopeful individuals recognized the inability to identify pathways and simple chose to set new goals and redirect their energy. Considering some evidence has pointed to hopefulness as being a predictor for mental health,^8,9,11,12^ future interventions aimed at students should strongly consider delivering positive coping strategies for this priority group, especially during times of crises and unexpected changes.

Hopefulness may be a learned behavior using positive psychology.^31^ Findings from our study revealed hope was negatively affected throughout the COVID-19 pandemic due to changes in students’ social and physical environments, suggesting the need for interventions may be amplified. Hope-based interventions have been delivered across different populations, including students, and have been shown to increase hope and, in some instances, also sustain high hope.^32–36^ These interventions generally emphasize one or more components of the following: goal-directed thinking, pathways to achieve goals, reframing potential obstacles to one’s goals, provision of support for maintaining hope, and visualization exercises.^32–34,37^ Some research studies have also found hope to increase ratings of life satisfaction, optimism, self-worth, and academic performance.^33,35,37^ The flexibility in the modality and duration of delivery of hope interventions make them a feasible type of intervention for university students which can benefit this group in multiple domains.^32,35,37^

Previously published research has found a connection between a sense of community and quality of life, hope, and subjective well-being.^38,39^ Not only are students with more robust social support less likely to struggle with mental-health-related issues,^40^ they are also more likely to be persistent,^41^ a trait our participants frequently linked to hopefulness. All participants from our study who lived on campus expressed their residence made them feel like they were part of a community due to the proximity to other students. However, the COVID-19 pandemic introduced several changes to people’s lives and postsecondary students were disproportionately affected by often having to move back home with parents. According to our findings, these eliminated many of the existing social supports and sense of community that students had developed during their academic experience. Previous research has demonstrated campus club involvement with higher levels of psychosocial development,^42^ and social support systems and hope have a protective effect on mental well-being.^12^ In addition, virtual interventions such as those delivered through smartphones have also been used to increase hope.^36^ These findings, along with our study, suggest universities should consider providing resources to campus organizations, mandate virtual campus activities to ensure students have access to various online and in-person communities, which can serve as avenues to maintain hope during times of crises.

Our findings revealed that most participants experienced loneliness, which may be an experience unique to the COVID-19 pandemic. Some evidence has suggested that hopelessness is associated with many aspects of worry, anxiety, and low socioeconomic status, and these factors may be exacerbated by the COVID-19 pandemic.^12,15^ Public health measures were taken to limit the spread of COVID-19 worldwide, and in many regions, this included transitioning to a remote learning environment. The challenges expressed by participants in our study include an increased number of smaller assessments, poor engagement in class, procrastination, and loneliness. This has been supported by another study assessing remote learning during the pandemic which found similar results suggesting a negative impact associated with distance education learning.^43^ However, distance education also provided some benefits to students in our study that supported identifying pathways to goals. The remote learning environment provides the pathways for students dealing with social anxiety and learning accommodations to succeed. While there were several disadvantages to remote learning from participants in our study, there were also some advantages. In fact, some studies examining remote learning have revealed that it can facilitate positive learning experience, satisfaction, and belongingness.^44,45^ There may also be advantages to implementing hybrid learning in the long term as studies have shown that mental health and student satisfaction with remote learning improve over time.^45^ Postsecondary institutions can consider alternative models to teaching and learning to reap the benefits along with traditional course delivery methods. We recommend universities attempt to incorporate some of the benefits of remote learning going forward and continue research in hybrid delivery programs to assess academic and mental health outcomes.

## Limitations

Despite the thick and rich description of data and student researcher-embedded values, this study was limited to participants from one postsecondary institution. Many of the participants in our study experienced loneliness and half of the participants had been diagnosed with depression, which is higher compared to the 2022 national average of 59% for loneliness and 25% for depression.^4^ The meanings of hope and interactions with hopefulness may be different in this group of individuals compared to a student population that is more representative based on clinically significant mental health outcomes. Furthermore, topics of hope and hopelessness were within the context of the COVID-19 pandemic. This provided a unique opportunity to explore COVID-19-era student challenges, but our findings may need to be replicated outside of the pandemic and in a cohort of students who are demographically different from this study to assess whether these perspectives change. Moreover, this study used videoconferencing software to interview participants. This naturally resulted in some unique setbacks such as breaks in connection and the loss of some nuances that in-person conversations contain (e.g., body language). However, we proceeded with virtual methods out of necessity during the COVID-19 pandemic and because there are other benefits such as allowing participants to be interviewed from their comfortable spaces and saving time on transportation.^46^ We also used member checking by inviting participants to comment on the study findings offline. As mentioned in previous study, there are risks to using member checks,^47^ and we attempted our best to avoid them by clearly communicating to participants the purpose of the member check and instructions on what the research team was looking for. Lastly, the topic of hopelessness can be sensitive to some, and it is possible that some student participants were not fully forthcoming or transparent. Although this did not diminish the interviews in this study because of the researcher-participant relationships and rapport, future iterations of these studies and other sensitive mental health topics could adopt telephone interviews and or chat-based data collection tools to ensure a safe space to share experiences of sensitive topics.

## Conclusions

Overall, hope was constructed based on behaviors modeled by others, feelings experienced within oneself, and associated with resilience. Furthermore, COVID-19 disrupted day-to-day life, and these disruptions to the social and physical environments exacerbated feelings of hopelessness. Our findings contribute to literature by providing insights to the effects of remote learning, the value of campus life, and the closely linked nature of hope with general mental health. This also provides a unique lens to the importance of hope in the context of coping strategies. Postsecondary institutions are advised to consider bolstering hope by delivering coping strategies and activities that enable it, investing and promoting virtual campus clubs in addition to traditional in-person extracurricular activities, and considering the introduction of hybrid teaching and learning methods for long term to enhance student satisfaction and mental health. These avenues may be a cost-effective means to improve student mental health across postsecondary institutions.

## Supplemental Material

sj-docx-1-smo-10.1177_20503121231185014 – Supplemental material for A qualitative study of university students’ perspectives of hope during the COVID-19 pandemic Click here for additional data file. Supplemental material, sj-docx-1-smo-10.1177_20503121231185014 for A qualitative study of university students’ perspectives of hope during the COVID-19 pandemic by Aria Keshoofy, Konrad Lisnyj, David L Pearl, Abhinand Thaivalappil and Andrew Papadopoulos in SAGE Open Medicine
