# Supplementary material for: A qualitative study of university students’ perspectives of hope during the COVID-19 pandemic
Source: SAGE Open Med. 2023 Jul 23;11:20503121231185014. doi: 10.1177/20503121231185014 (PMC10366665; doi:10.1177/20503121231185014)
Supplement: sj-docx-1-smo-10.1177_20503121231185014 – Supplemental material for A qualitative study of university students’ perspectives of hope during the COVID-19 pandemic [file sj-docx-1-smo-10.1177_20503121231185014.docx]

**Interview Guide and Demographic Survey for Hope during the COVID-19 Pandemic Study**

**Section A: Interview guide**

*The purpose of this interview is to engage in a 30–45-minute discussion with postsecondary students to identify their perceptions of hope, hopelessness, and how the last 12 months have impacted their life. Interviews will be one-on-one online and will be semi-structured. Because of this, the exact wording may change. Sometimes, we will use other short probing questions to make sure we understand what you told me or if we need more information when we are talking, such as “please tell me more,” or “why do you think that?”*

1. In general, what comes to mind when you think of the term “hope”?
2. How would you describe a person who is hopeless? What specific characteristics might be synonymous with a hopeless person?
3. Can you think of anyone in your life that you look up to that could be described as hopeful? If so, what about them gives off this aura?
4. Before March 2020, would you have described yourself as a hopeful person? Why or why not? And did this change after the pandemic began? When an unexpected adverse event occurs, how do you respond?
5. In general, would you describe yourself as a hopeful person now? What about prior to COVID-19 pandemic? What about now?
6. [Only for students who lived on campus] Did you enjoy residence? Would you say it was community oriented? Did it give you a welcoming feeling to be there? What could have been improved?
7. In the last 12 months, with lockdowns and different levels of “opening up” businesses, how much has your normal life changed throughout the COVID-19 pandemic? What was a typical day in the life like prior to COVID-19? What does that look like now? What about social interactions?
8. Referring to the previous question, how has this impacted your mental health?
9. How has online learning impacted your life? Did you enjoy it? Why or why not? Was online course delivery different? Did it affect your loneliness?
10. How has being a student impacted your mental health? What are the greatest stressors that university has put on you? If you consider yourself a hopeful person, what role has being hopeful played in this? If not, what can the university do to improve this?
11. Are you a part of any clubs or teams on campus? How has this impacted your mental health? What about in the community? Is there any kind of groups you belong to?
12. When would you say you are truly happiest? Please explain.
13. When would you say you are the least happy? Please explain.
14. Imagine a hypothetical situation where there is one individual in charge of all aspects of the university, if you had a chance to talk with them, what is one thing you would you ask them to change on campus to improve your mental health?
15. Is there anything at all that you would like to add?

**Section B: Demographic characteristics**

*We are interested in obtaining some additional information that will provide context to the study findings. This information will be anonymous and confidential and will not be linked to you. Please highlight the responses below and return this document to us.*

1. What is your gender identity?
   1. Woman
   2. Man
   3. My gender is not listed above, please specify: _____
   4. Choose not to respond
2. What is your approximate cumulative GPA?
   1. A
   2. B
   3. C
   4. D/F
   5. Not applicable
   6. Choose not to respond
3. What college or faculty do you belong to?
   1. Arts
   2. Biological Science
   3. Business
   4. Engineering and Physical Sciences
   5. Social and Applied Human Sciences
   6. Ontario Agricultural College
   7. Ontario Veterinary College
4. How would you describe your general health?
   1. Excellent
   2. Very good
   3. Good
   4. Fair
   5. Poor
   6. Don’t know
5. In the last 12 months, have you ever felt very lonely?
   1. No, never
   2. No, not in the last 12 months
   3. Yes, in the last 2 weeks
   4. Yes, in the last 30 days
   5. Yes, in the last 12 months
   6. Choose not to respond
6. In the last 12 months, have you ever seriously considered suicide?
   1. No, never
   2. No, not in the last 12 months
   3. Yes, in the last 2 weeks
   4. Yes, in the last 30 days
   5. Yes, in the last 12 months
   6. Choose not to respond
7. In the last 12 months, have you ever attempted suicide?
   1. No, never
   2. No, not in the last 12 months
   3. Yes, in the last 2 weeks
   4. Yes, in the last 30 days
   5. Yes, in the last 12 months
   6. Choose not to respond
8. Have you ever been diagnosed with depression?
   1. No
   2. Yes
   3. Choose not to respond
9. Within the last 12 months, has financial stress been traumatic or very difficult for you to handle?
   1. No
   2. Yes
   3. Choose not to respond
10. Within the last 12 months, has academics been traumatic or very difficult for you to handle?
    1. No
    2. Yes
    3. Choose not to respond
11. Within the last 12 months, has finances been traumatic or very difficult for you to handle?
    1. No
    2. Yes
    3. Choose not to respond
12. In the past month, how often did you have the feeling that you belonged to a community like a social group or your neighborhood?
    1. Never
    2. Once or twice
    3. About once a week
    4. About two or three times a week
    5. Almost everyday
    6. Everyday
